# Bilateral Mandibular Supernumerary Canines: A Case Report

**DOI:** 10.5681/joddd.2010.034

**Published:** 2010-12-21

**Authors:** Ehsan Abouei Mehrizi, Hassan Semyari, Gholamreza Eslami Amirabadi

**Affiliations:** ^1^ Post-graduate Student, Deptartment of Orthodontics, Faculty of Dentistry, Shahed University, Tehran, Iran; ^2^ Assistant Professor, Deptartment of Periodontics, Faculty of Dentistry, Shahed University, Tehran, Iran; ^3^ Assistant Professor, Deptartment of Orthodontics, Faculty of Dentistry, Shahed University, Tehran, Iran

**Keywords:** Canine, supernumerary, tooth

## Abstract

Supernumerary teeth are defined as the teeth developed in excess of the number found in a normal dentition. Supernumerary canine is an extremely rare finding particularly in the mandible. This case report presents a 25-year-old female patient with the unique feature of bilateral mandibular supplemental supernumerary canines. The patient was non-syndromic without any other supernumerary teeth.

## Introduction

Supernumerary teeth are defined as extra teeth that develop in addition to the normal dental formula.^1^ Most clinical complications associated with supernumerary teeth are related to interference with normal eruption of the adjacent teeth that can result in retarded eruption, impaction or malalignment.^2^ Approximately 75% of supernumerary teeth are impacted, and are mostly diagnosed coincidentally during radiographic examination.^3^ Impacted supernumerary teeth may lead to displacement of the adjacent teeth as well as root resorption and formation of dentigerous cysts.^4^

Supernumerary teeth may occur as isolated dental findings or as part of a syndrome. In the general Caucasian population, the prevalence of supernumerary teeth is 1-3% with slightly higher rates in Asian populations.^5^ Males are affected approximately twice as frequently as females.^4^

Supernumerary teeth may occur in any region of the dental arch,^6^ with 90-98% of occurrences in the maxilla.^4^ 90% of supernumerary teeth occur in the premaxilla region, 93% of which are in the central incisor region and 25% located in the midline. Of the other 10%, about 4% are located in the mandibular premolar and 1.5% in the maxillary canine regions.^7^

Single supernumerary teeth include 76-86% of all cases; 12-23% occur in pairs and multiple supernumerary teeth (i.e. three or more) has been found in less than 1% of cases.^4^

Based on the morphology supernumerary teeth are classified into four different types: (I) conical, (II) tuberculate, (III) odontoma and (IV) supplemental which has normal shape and size. In the study of Rajab & Hamdan,^7^ the prevalence of normal crown shape was 6.9%.

A review of the literature indicates that supernumerary central and lateral incisors and premolars have been observed frequently. Supernumerary canines, however, are extremely rare and are detected more often in the maxilla than in the mandible. In a research conducted among 1700 American adolescents, 64 supernumerary teeth were found, and none of them were supernumerary canines.^8^ Among 2241 Mexican children, only one had a supernumerary canine.^9^ In a study involving 152 Jordanian children with 202 supernumerary teeth, the prevalence of maxillary and mandibular supernumerary canines was 1.5% and 1%, respectively.^7^ The corresponding figures in a Brazilian study of 460 supernumerary teeth were 2.6 and 1.3% for maxilla and mandible, respectively.^10^

Based on the search conducted in the PubMed, with the exception of rare cases of multiple supernumerary teeth, a non-syndrome case of bilateral mandibular supplemental canines does not exist in the literature. The present paper reports a case of bilateral mandibular supernumerary canines.

## Case Report

**Figure 1:**
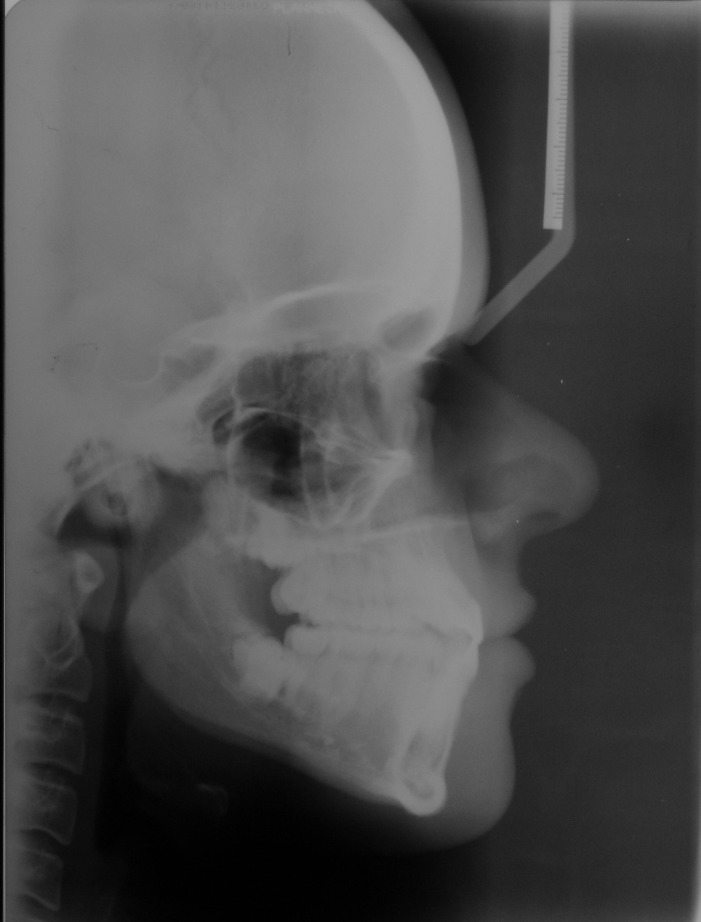
Lateral cephalometry.

A 25-year-old female patient was referred to the Department of Orthodontics, Dental School, Shahed University of Medical Sciences, Tehran, Iran. Past medical history revealed a kidney cyst, but the patient did not suffer from any systemic disease. Clinical examinations eliminated the possibilities of any developmental disorders and syndromes. Extra-oral examination did not reveal any abnormality. In intraoral examination, mild spacing in both of dental arches was noted. Her upper left lateral incisor, second premolar and first molar were in dental crossbite without any functional shift. She had class III occlusal relationships and cephalometric evaluation revealed a class III skeletal pattern (Figure 1). In her panoramic radiograph, four impacted third molars were present (Figure 2). Significant root dilaceration of maxillary lateral incisors and right first premolar was evident. Surprisingly, an impacted supernumerary canine was noted on the right side of the mandible and a primary canine accompanied by two impacted teeth, both resembling a canine, was present on the left side of the mandible. All of the three impacted teeth had fully-developed roots, and showed the configuration, size and root length of a permanent canine (Figures 2 & 3). Occlusal radiograph revealed the impacted tooth on the right was lingual and both of impacted teeth on the left were buccal (Figure 4). According to the patient, there was no history of tooth extraction. The parents did not have supernumerary teeth; the patient had no siblings.

 Figure 2. Panoramic radiograph (A) and periapical radiographs of mandibular canine region (B & C) before treatment.

**A:**
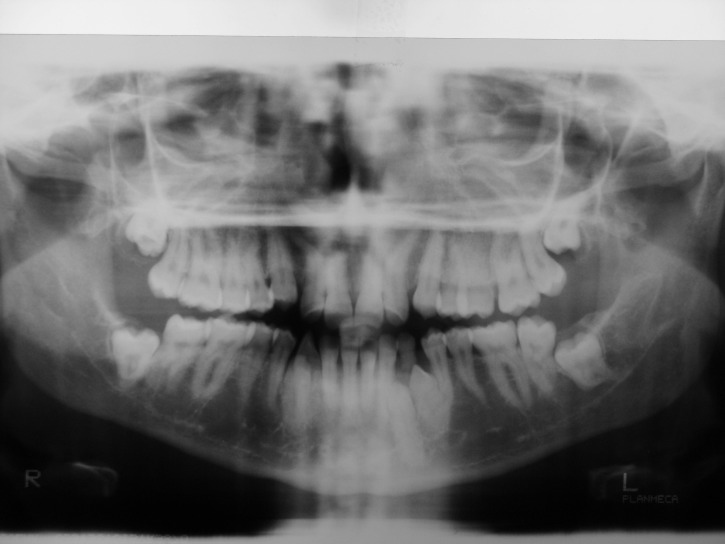

**B & C:**
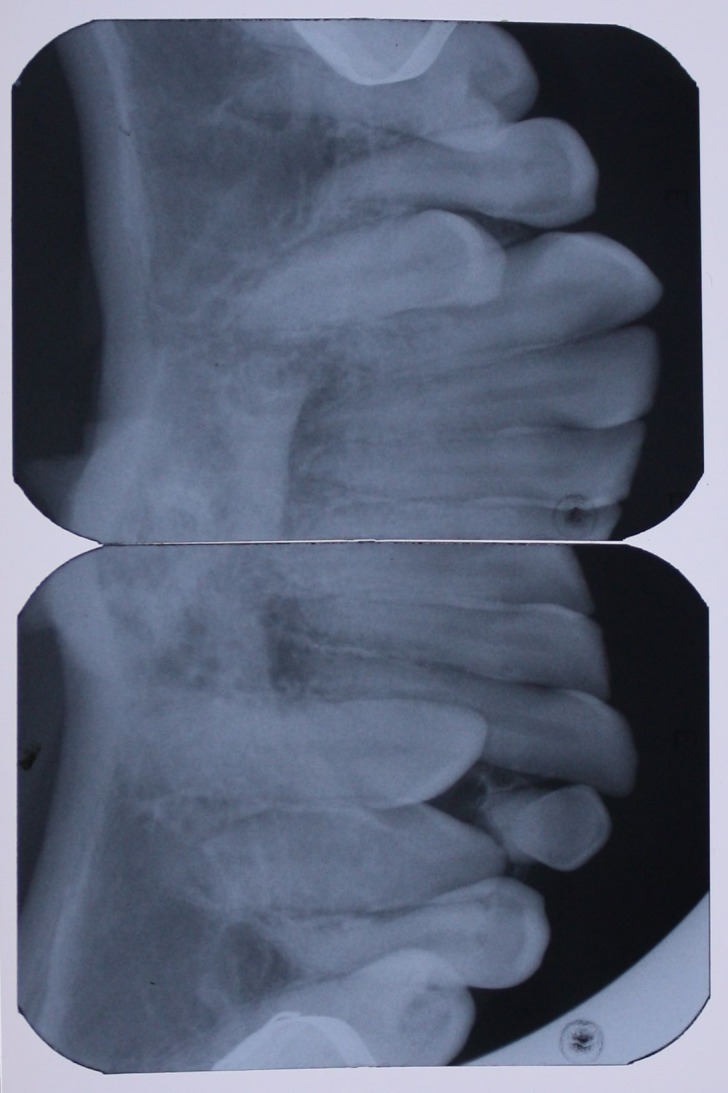


Figure 3. Panoramic radiograph (A) and periapical radiograph of mandibular left canine region (B) after the surgical removal of right supernumerary canine.

**A:**
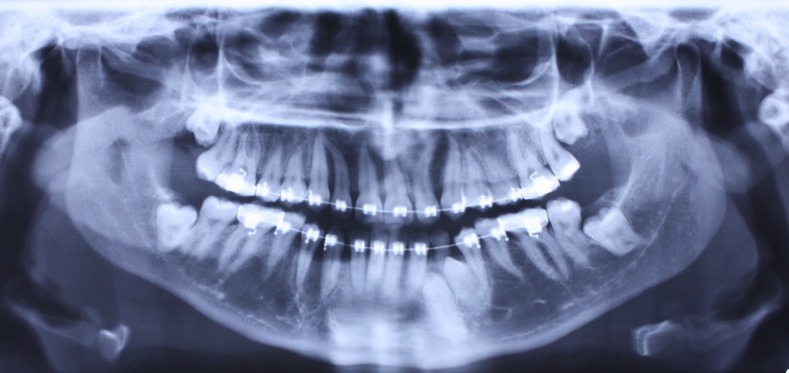

**B:**
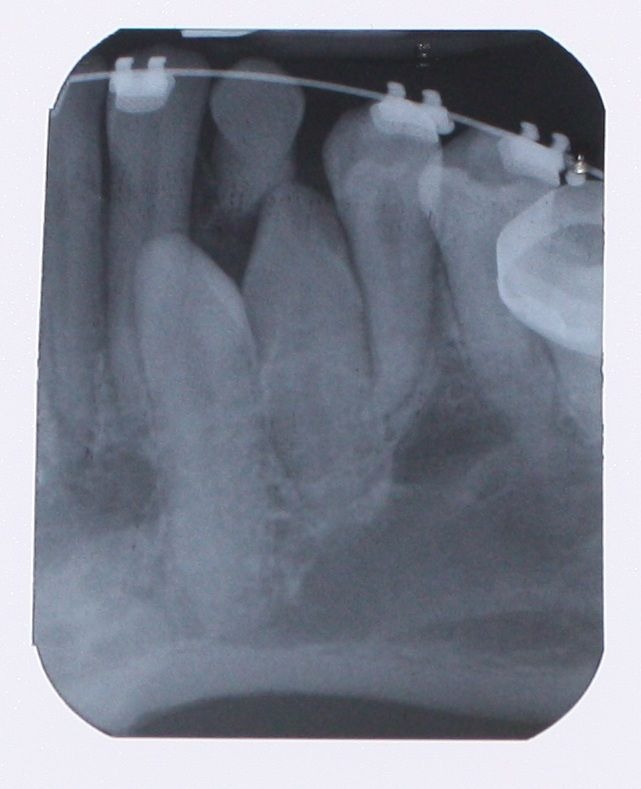


Figure 4. Occlusal view (A) and occlusal radiograph (B) of mandible before treatment, and occlusal radiograph (C) after leveling and aligning.

**A:**
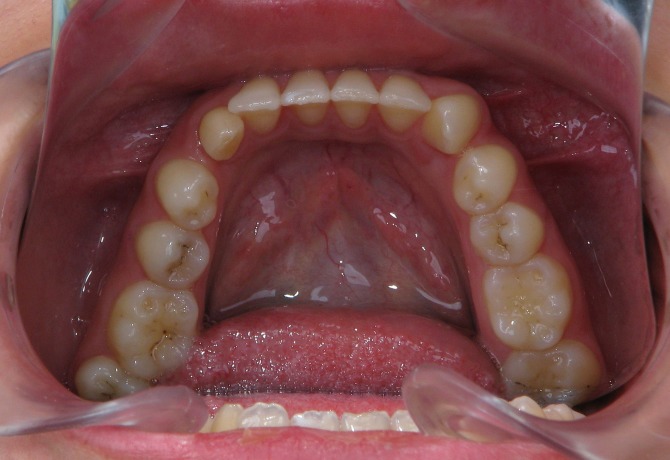

**B:**
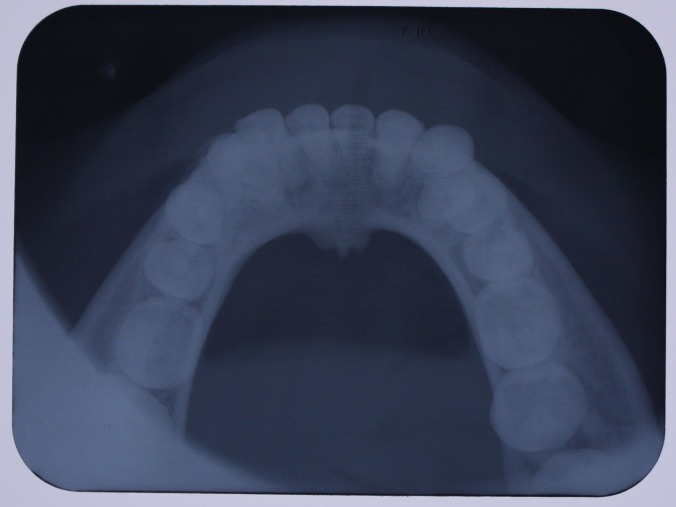

**C:**
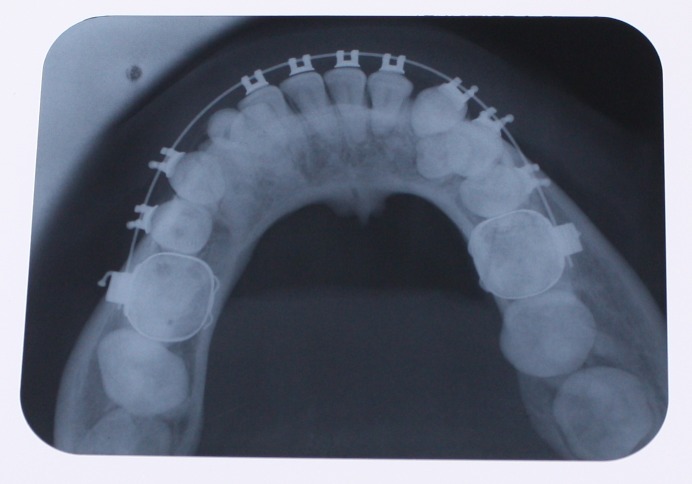

Fixed orthodontic treatment started for the patient. After six months of treatment including leveling and aligning, the right canine and first premolar were not properly aligned because of the impacted supernumerary canine; therefore, surgical removal of the supernumerary tooth was carried out.

Another surgical session was planned for the left side, in which the distal impacted canine was removed after extraction of the ankylosed primary canine, and a bracket was placed on the mesial canine for forced eruption because of its longer root. Both of the extracted teeth had the size and anatomy of a normal canine (Figure 5).


Figure 5. In second surgical session on the left side, it was planned to bracket the mesial and extract canine the distal one (A) supernumerary canine and ankylosed primary canine (B & C).

**A:**
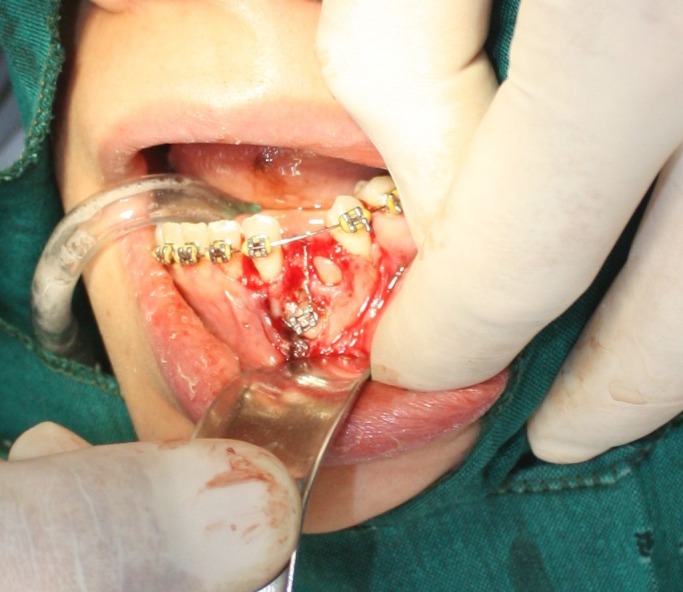

**B:**
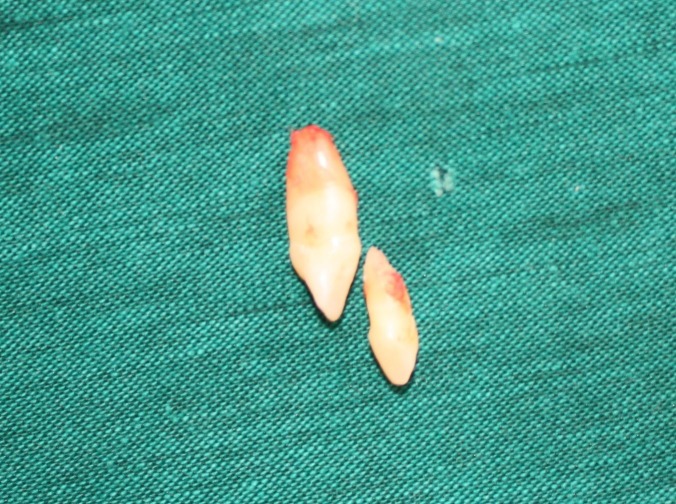

**C:**
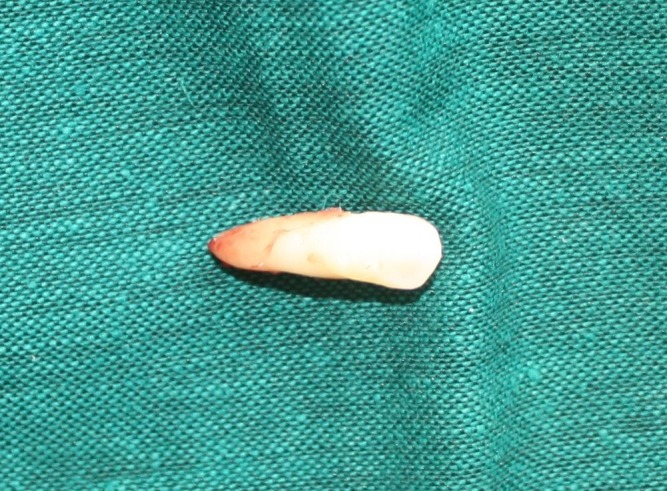

## Discussion

There have been several reports of bilateral supernumerary maxillary canines in the literature. Türkkahraman et al^6^ reported a case, and Cho et al^11^ described two cases of bilateral supernumerary maxillary canines. Sasaki et al^2^ reported a patient with four supernumerary teeth; bilateral supernumerary maxillary canines and supernumeraries in the mandibular canine-premolar region. Santos et al^12^ also reported a case of bilateral supernumerary maxillary canines accompanying supernumerary maxillary primary canines.

On the other hand, only two reports of bilateral mandibular supernumerary canines in subjects with multiple supernumerary teeth were found in the literature. Joshi^13^ presented a case with nine supernumerary teeth in an Indian 23-year-old male; located in the premolar and canine region of both jaws including two supernumerary mandibular canines. In addition, Hume^14^ described a case with four supernumerary canines, two in the maxilla and two in the mandible.

The etiology of supernumerary teeth as an isolated dental finding is not clearly understood. Heredity is an important etiologic factor,^7^ because of reports of occurrence within the same family.^15^ Supernumerary teeth seem to be polygenic in most instances.^4^ The presence of identical supernumerary teeth in monozygotic twins indicates the role of genetics.^16^The exact mode of inheritance has not been identified. Many cases suggest an autosomal dominant pattern of inheritance with incomplete penetrance. The variable expression and penetrance may be caused by environmental factors.^5^ Generally, it is believed environmental factors along with heredity contribute to the etiology.^17^

Numerous developmental disorders also are associated with hyperdontia; cleft lip and palate,^6^ and the following syndromes: Apert, Angio-osteohyper-trophy, cleidocranial dysplasia, craniometaphyseal dysplasia, Crouzon, Curtius, Down, Ehlers-Danlos, Fabry-Anderson, Fucosidosis, Gardner, Hallermann-Streiff, Klippel-Trenaunay-Weber, Laband, Nance-Horan, Oral-facial-digital type I and III, Sturge-Weber, and Tricho-rhino-phalangeal.^5^ Defined Mutations are associated with the cleidocranial dysplasia, Gardner’s and Nance-Horan syndromes,^18^ but no mutations in association with non syndromic supernumerary teeth have been identified yet.^19^

Several theories have been suggested to explain hyperdontia. One theory is phylogenic process of atavism.^15^ Another popularly accepted theory is the dichotomy theory of tooth germs, according to which the tooth bud split into two equal or different sized parts, resulting in two teeth of equal size or one normal and one dismorphic tooth.^6^ This hypothesis is supported by animal experiments in which split tooth germs have been cultivated in vitro.^20^. According to the most accepted theory, supernumerary teeth are formed as a result of local, independent, or conditioned hyperactivity of dental lamina.^15^

Several researchers have proposed multiple supernumerary teeth are part of a post permanent dentition.^3,7,21^

For patients previously diagnosed with supernumerary teeth or those genetically predisposed, long-term monitoring for additional supernumerary tooth development is recommended.

## References

[1] Dummett CO, Jr. Anomalies of the developing dentition. In: Pinkham JR, ed. Pediatric Dentistry: Infancy through Adolescence, 3rd ed. Philadelphia: W.B. Saunders; 1999: 43–5.

[2] Sasaki H, Funao J, Morinaga H, Nakano K, Ooshima T (2007). Multiple supernumerary teeth in the maxillary canine and mandibular premolar regions: a case in the postpermanent dentition. Int J Paediatr Dent.

[3] Açikgöz A, Açikgöz G, Tunga U, Otan F (2006). Characteristics and prevalence of non-syndrome multiple supernumerary teeth: a retrospective study. Dentomaxillofac Radiol.

[4] So LL (1990). Unusual supernumerary teeth. Angle Orthod.

[5] Neville BW, Damm DD, Allen CM, Bouquot JE. Oral & Maxillofacial Pathology, 2nd ed. Philadelphia: WB Saunders; 2002: 69-73.

[6] Türkkahraman H, Yilmaz HH, Cetin E (2005). A non-syndrome case with bilateral supernumerary canines: report of a rare case. Dentomaxillofac Radiol.

[7] Rajab LD, Hamdan MA (2002). Supernumerary teeth: review of the literature and a survey of 152 cases. Int J Paediatr Dent.

[8] Harris EF, Clark LL (2008). An epidemiological study of hyperdontia in American blacks and whites. Angle Orthod.

[9] Salcido-García JF, Ledesma-Montes C, Hernández-Flores F, Pérez D, Garcés-Ortíz M (2004). Frequency of supernumerary teeth in Mexican population. Med Oral Patol Oral Cir Bucal.

[10] De Oliveira Gomes, Drummond SN, Jham BC, Abdo EN, Mesquita RA (2008). A survey of 460 supernumerary teeth in Brazilian children and adolescents. Int J Paediatr Dent.

[11] Cho SY, Yeung KH, Lee CK (2007). Supplemental permanent maxillary canines: report of two rare bilateral cases. Prim Dent Care.

[12] Santos AP, Ammari MM, Moliterno LF, Júnior JC (2009). First report of bilateral supernumerary teeth associated with both primary and permanent maxillary canines. J Oral Sci.

[13] Joshi MR (1966). Mandibular supernumerary canines in a patient with multiple supernumerary teeth: report of a case. Oral Surg Oral Med Oral Pathol.

[14] Hume WJ (1973). Supplemental canines. A case report. J Dent.

[15] Marya CM, Kumar BR (1998). Familial occurrence of mesiodentes with unusual findings: case reports. Quintessence Int.

[16] Langowska-Adamczyk H, Karmańska B (2001). Similar locations of impacted and supernumerary teeth in monozygotic twins: a report of 2 cases. Am J Orthod Dentofacial Orthop.

[17] Brook AH (1984). A unifying aetiological explanation for anomalies of human tooth number and size. Arch Oral Biol.

[18] Bailleul-Forestier I, Berdal A, Vinckier F, de Ravel T, Fryns JP, Verloes A (2008). The genetic basis of inherited anomalies of the teeth. Part 2: syndromes with significant dental involvement. Eur J Med Genet.

[19] Bailleul-Forestier I, Molla M, Verloes A, Berdal A (2008). The genetic basis of inherited anomalies of the teeth. Part 1: clinical and molecular aspects of non-syndromic dental disorders. Eur J Med Genet.

[20] Gündüz K, Muğlali M (2007). Non-syndrome multiple supernumerary teeth. A case report. J Contemp Dent Pract.

[21] Becker A, Bimstein E, Shteyer A (1982). Interdisciplinary treatment of multiple unerupted supernumerary teeth: report of a case. Am J Orthod.

